# Supplementary material for: pH and Salt-Assisted Macroscopic Chirality Inversion of Gadolinium Coordination Polymer
Source: Molecules. 2022 Dec 25;28(1):163. doi: 10.3390/molecules28010163 (PMC9821918; doi:10.3390/molecules28010163)
Supplement: Supplementary file 1 [file molecules-28-00163-s001.zip › molecules-2083990-supplementary.pdf]

Supporting Information for

pH and Salt-Assisted Macroscopic Chirality Inversion of Gadolinium Coordination Polymer

Ting Hou, [1] Lan-Qing Wu, [1] Yan Xu, [1,2] Song-Song Bao, [1] and Li-Min Zheng [1,*]

[1]State Key Laboratory of Coordination Chemistry, School of Chemistry and Chemical Engineering, Collaborative Innovation Center of Advanced Microstructures, Nanjing University, Nanjing, 210023, China

[2]Institute of Information Engineering, Suqian College, Suqian 223800, China

*Correspondence: lmzheng@nju.edu.cn

Content

Ⅰ. pH effect on the reaction product 3

Ⅱ. Structures and characterization of ***R*-Block** 6

Ⅲ. Characterization of superhelices ***R*-M** and ***R*-P** 12

Ⅳ. Salt effect on the chirality inversion of superhelices 15

Ⅰ. pH effect on the reaction product

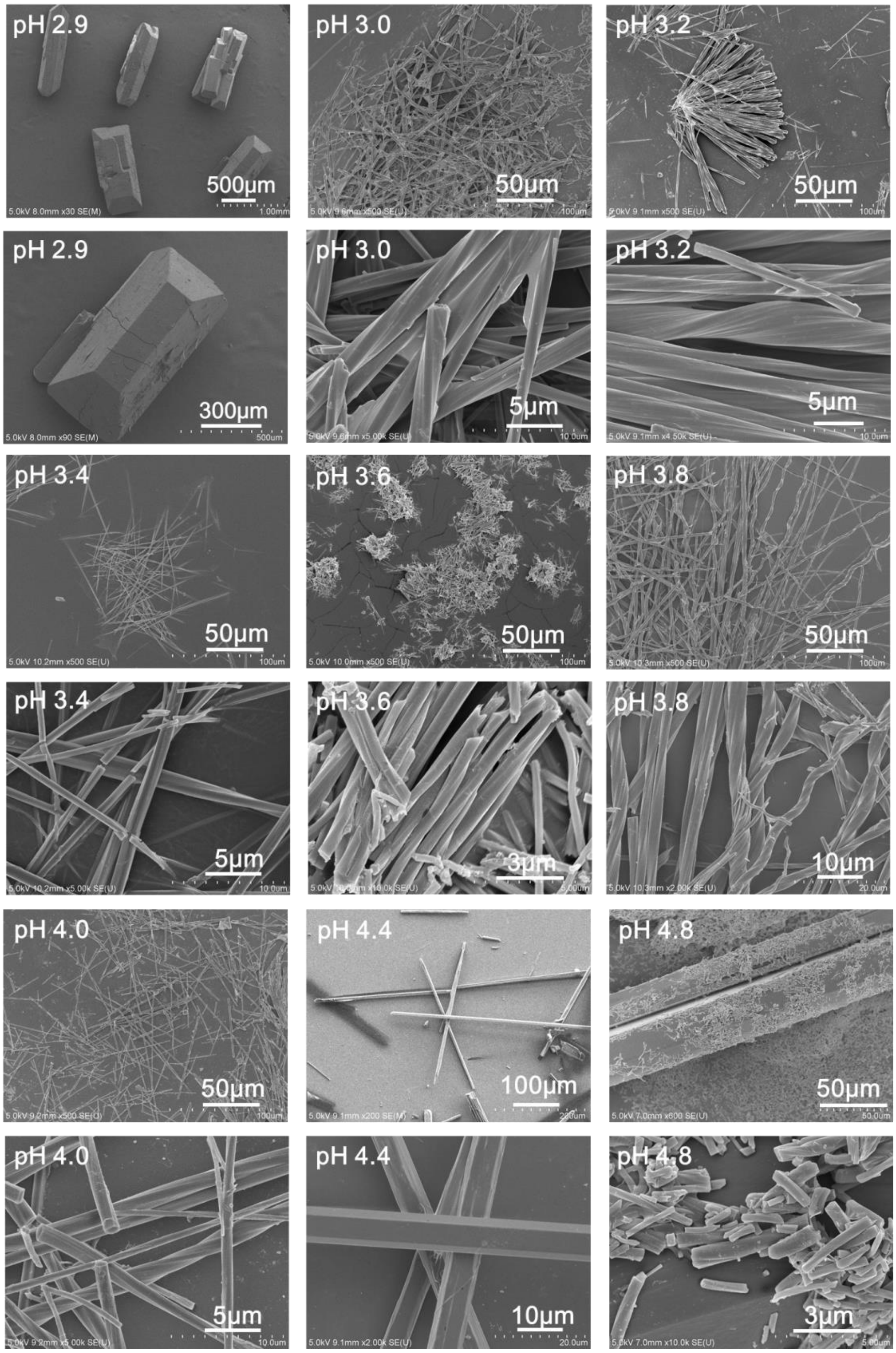

Figure S1. SEM images show the morphology of the product under different pH of mixture after hydrothermal reactions of $Gd(NO_3)_3$ and *R*-pempH_2 at 120°C for 24h.

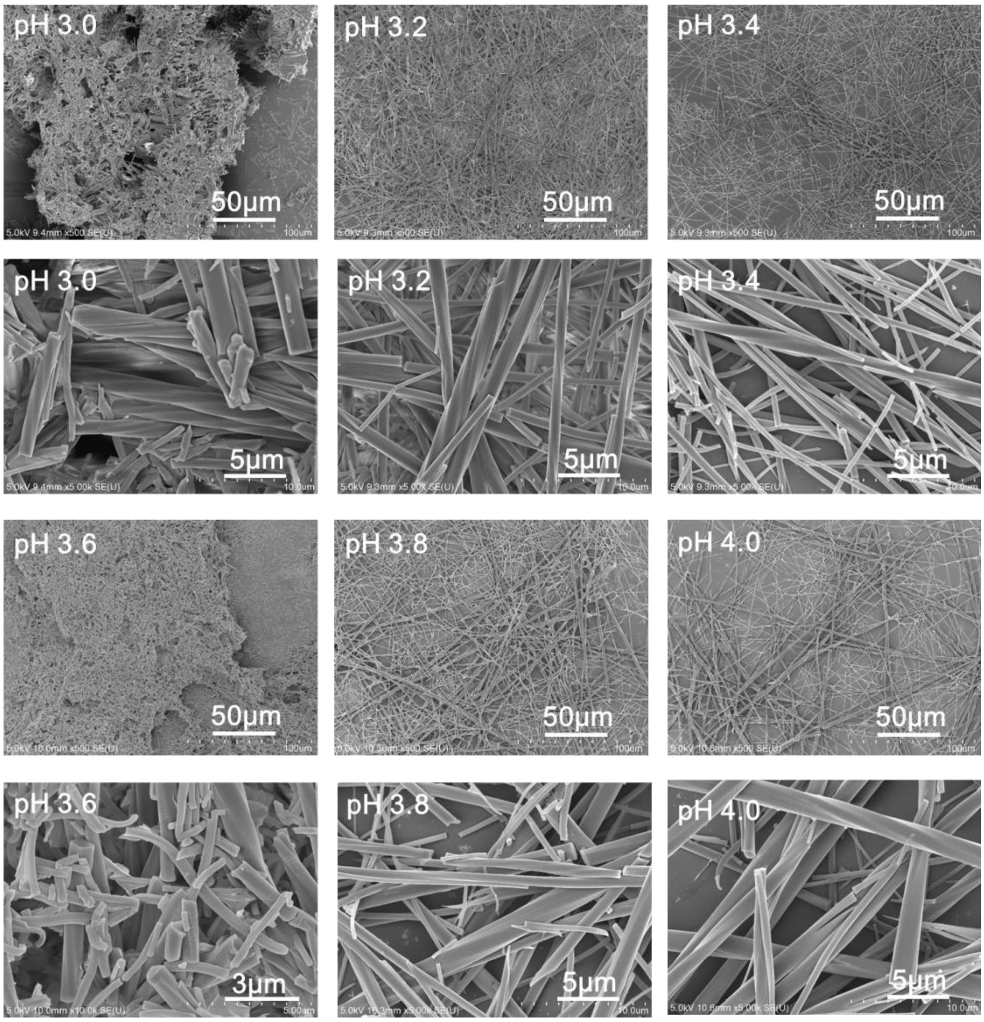

Figure S2. SEM images show the morphology of the product under different pH of mixture after hydrothermal reactions of $Tb(NO_3)_3$ and *R*-pempH$_2$ at 120°C for 24h.

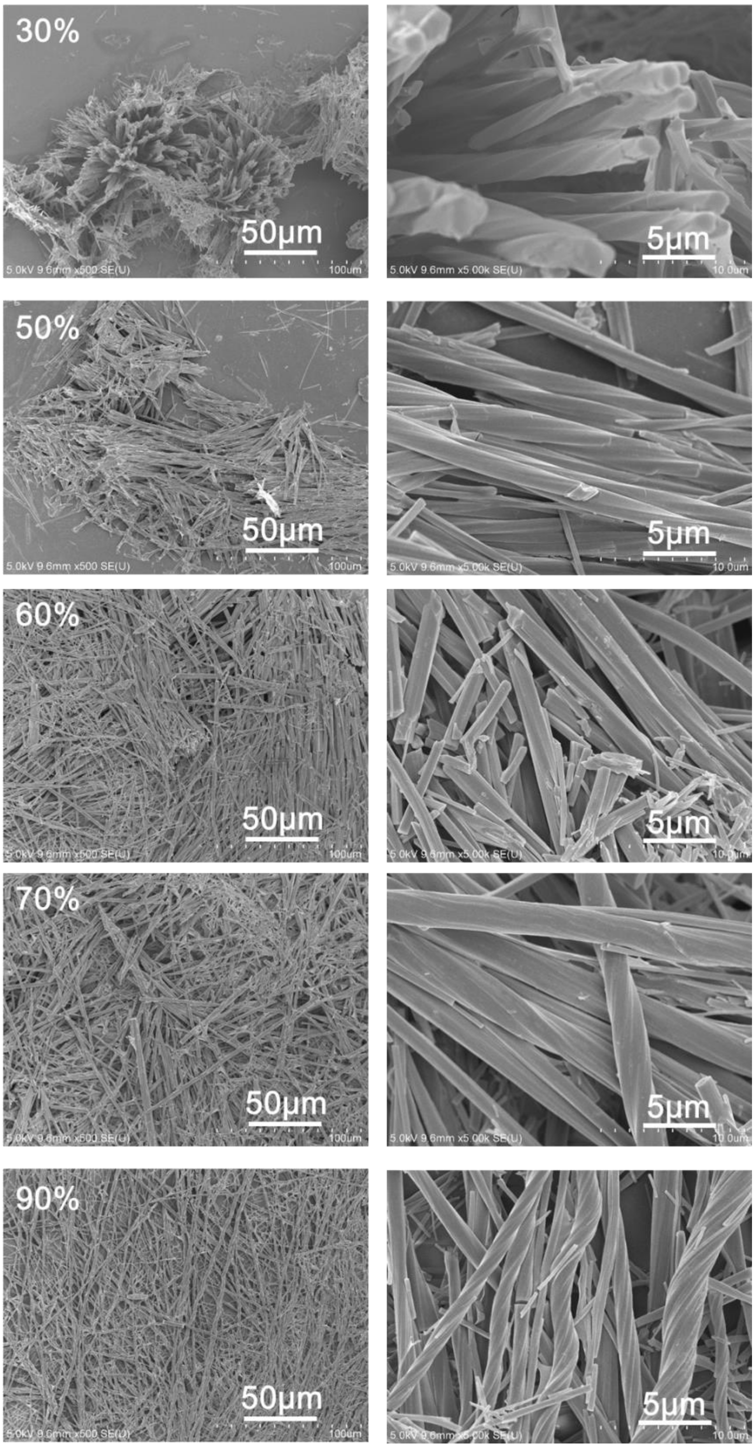

Figure S3. SEM images show the product under different loading coefficient (30%; 50%; 60%; 70%; 90%) after hydrothermal reactions of $Gd(NO_3)_3$ and *R*-pempH_2 at pH 3.2 and 120°C for 24h.

Ⅱ. Structures and characterization of *R*-Block

Table S1. Crystal data and structure refinements for ***R*-Block**.

**Compounds**	***R*-Block**
Formula	$C_{162}H_{262}Gd_6N_{22}O_{78}P_{18}$
M	5266.88
Crystal size [mm]	0.40 x 0.25 x 0.25
Crystal system	monoclinic
Space group	$P2_1$
T(K)	193(2) K
a (Å)	17.1065(16)
b (Å)	24.209(2)
c (Å)	26.190(3)
α (°)	90
β (°)	92.4336(15)
γ (°)	90
V (Å^3)	10836.0(18)
D_c (g cm^{-3})	1.614
μ (mm^{-1})	2.033
F (000)	5332
R_{int}	0.0402
T_{max}, T_{min}	0.7455, 0.5414
GoF on F^2	1.017
R_1 ,wR_2 [I>2σ(I)]	0.0374, 0.0774
(all data)	0.0453, 0.0824
Flack parameter	-0.017(3)
$(\Delta\rho)_{max,min}$/e Å^{-3}	1.649, -0.744
CCDC number	2086976

Table S2. Selected bond lengths [Å] and angles [˚] for ***R*-Block**.

Gd(1)-O(1)	2.369(5)	Gd(4)-O(40)	2.305(5)
Gd(1)-O(8)	2.396(5)	Gd(4)-O(28)	2.312(5)
Gd(1)-O(20)#1	2.403(5)	Gd(4)-O(34)	2.314(5)
Gd(1)-O(24)#1	2.444(5)	Gd(4)-O(53)#2	2.335(5)
Gd(1)-O(26)#1	2.448(5)	Gd(4)-O(31)	2.397(5)
Gd(1)-O(7)	2.499(5)	Gd(4)-O(37)	2.475(5)
Gd(1)-O(22)#1	2.533(5)	Gd(4)-O(38)	2.476(5)
Gd(2)-O(5)	2.310(5)	Gd(5)-O(35)	2.293(5)
Gd(2)-O(16)	2.331(5)	Gd(5)-O(49)	2.327(5)
Gd(2)-O(13)	2.353(5)	Gd(5)-O(38)	2.344(5)
Gd(2)-O(7)	2.365(5)	Gd(5)-O(43)	2.379(5)
Gd(2)-O(2)	2.393(5)	Gd(5)-O(41)	2.423(5)
Gd(2)-O(11)	2.435(5)	Gd(5)-O(46)	2.451(5)
Gd(2)-O(1)	2.543(5)	Gd(5)-O(47)	2.463(5)
Gd(2)-O(10)	2.631(5)	Gd(5)-O(40)	2.525(5)
Gd(3)-O(10)	2.345(5)	Gd(6)-O(44)	2.328(5)
Gd(3)-O(22)	2.350(5)	Gd(6)-O(32)#3	2.329(5)
Gd(3)-O(25)	2.367(5)	Gd(6)-O(47)	2.336(5)
Gd(3)-O(19)	2.387(5)	Gd(6)-O(29)#3	2.338(5)
Gd(3)-O(14)	2.408(5)	Gd(6)-O(52)	2.388(5)
Gd(3)-O(17)	2.417(5)	Gd(6)-O(49)	2.449(5)
Gd(3)-O(13)	2.508(5)	Gd(6)-O(50)	2.469(5)
Gd(3)-O(20)	2.600(5)	Gd(6)-O(53)	2.576(5)
O(4)-Gd(1)-O(8)	104.79(18)	O(14)-Gd(3)-O(20)	90.74(16)
O(1)-Gd(1)-O(8)	125.67(16)	O(17)-Gd(3)-O(20)	141.38(16)
O(4)-Gd(1)-O(20)#1	82.48(17)	O(13)-Gd(3)-O(20)	142.04(15)
O(1)-Gd(1)-O(20)#1	154.48(17)	O(40)-Gd(4)-O(28)	83.84(17)
O(8)-Gd(1)-O(20)#1	75.73(16)	O(40)-Gd(4)-O(34)	90.03(17)
O(4)-Gd(1)-O(24)#1	152.89(17)	O(28)-Gd(4)-O(34)	88.85(19)
O(1)-Gd(1)-O(24)#1	76.04(17)	O(40)-Gd(4)-O(53)#2	165.76(17)
O(8)-Gd(1)-O(24)#1	82.73(18)	O(28)-Gd(4)-O(53)#2	82.28(17)
O(20)#1-Gd(1)-O(24)#1	124.57(17)	O(34)-Gd(4)-O(53)#2	86.55(18)
O(4)-Gd(1)-O(26)#1	86.95(17)	O(40)-Gd(4)-O(31)	78.07(17)
O(1)-Gd(1)-O(26)#1	79.91(16)	O(28)-Gd(4)-O(31)	90.70(19)
O(8)-Gd(1)-O(26)#1	153.14(16)	O(34)-Gd(4)-O(31)	168.08(17)
O(20)#1-Gd(1)-O(26)#1	82.14(16)	O(53)#2-Gd(4)-O(31)	105.20(17)
O(24)#1-Gd(1)-O(26)#1	97.85(18)	O(40)-Gd(4)-O(37)	118.80(17)
O(4)-Gd(1)-O(7)	76.46(17)	O(28)-Gd(4)-O(37)	149.92(17)
O(1)-Gd(1)-O(7)	69.54(16)	O(34)-Gd(4)-O(37)	109.25(18)
O(8)-Gd(1)-O(7)	59.39(16)	O(53)#2-Gd(4)-O(37)	75.32(17)
O(20)#1-Gd(1)-O(7)	122.20(16)	O(31)-Gd(4)-O(37)	76.39(17)
O(24)#1-Gd(1)-O(7)	85.77(17)	O(40)-Gd(4)-O(38)	69.99(15)

O(26)#1-Gd(1)-O(7)	147.43(16)	O(28)-Gd(4)-O(38)	151.19(17)
O(4)-Gd(1)-O(22)#1	145.79(16)	O(34)-Gd(4)-O(38)	79.60(16)
O(1)-Gd(1)-O(22)#1	119.26(16)	O(53)#2-Gd(4)-O(38)	122.75(16)
O(8)-Gd(1)-O(22)#1	88.36(16)	O(31)-Gd(4)-O(38)	95.30(16)
O(20)#1-Gd(1)-O(22)#1	70.17(16)	O(37)-Gd(4)-O(38)	58.39(15)
O(24)#1-Gd(1)-O(22)#1	58.61(16)	O(35)-Gd(5)-O(49)	78.72(17)
O(26)#1-Gd(1)-O(22)#1	69.70(16)	O(35)-Gd(5)-O(38)	78.37(17)
O(7)-Gd(1)-O(22)#1	135.61(15)	O(49)-Gd(5)-O(38)	155.72(16)
O(5)-Gd(2)-O(16)	85.58(19)	O(35)-Gd(5)-O(43)	83.29(17)
O(5)-Gd(2)-O(13)	80.73(17)	O(49)-Gd(5)-O(43)	89.62(17)
O(16)-Gd(2)-O(13)	80.57(18)	O(38)-Gd(5)-O(43)	79.82(16)
O(5)-Gd(2)-O(7)	84.26(17)	O(35)-Gd(5)-O(41)	112.31(18)
O(16)-Gd(2)-O(7)	81.48(17)	O(49)-Gd(5)-O(41)	74.71(16)
O(13)-Gd(2)-O(7)	157.37(18)	O(38)-Gd(5)-O(41)	121.95(16)
O(5)-Gd(2)-O(2)	103.16(17)	O(43)-Gd(5)-O(41)	154.50(16)
O(16)-Gd(2)-O(2)	156.15(17)	O(35)-Gd(5)-O(46)	158.12(16)
O(13)-Gd(2)-O(2)	79.06(17)	O(49)-Gd(5)-O(46)	123.12(16)
O(7)-Gd(2)-O(2)	121.09(17)	O(38)-Gd(5)-O(46)	79.84(16)
O(5)-Gd(2)-O(11)	159.37(16)	O(43)-Gd(5)-O(46)	94.78(17)
O(16)-Gd(2)-O(11)	95.48(19)	O(41)-Gd(5)-O(46)	78.01(17)
O(13)-Gd(2)-O(11)	119.81(18)	O(35)-Gd(5)-O(47)	138.74(17)
O(7)-Gd(2)-O(11)	75.56(17)	O(49)-Gd(5)-O(47)	68.85(17)
O(2)-Gd(2)-O(11)	84.08(17)	O(38)-Gd(5)-O(47)	126.67(17)
O(5)-Gd(2)-O(1)	72.61(16)	O(43)-Gd(5)-O(47)	71.99(16)
O(16)-Gd(2)-O(1)	144.37(17)	O(41)-Gd(5)-O(47)	83.50(16)
O(13)-Gd(2)-O(1)	121.59(16)	O(46)-Gd(5)-O(47)	59.16(16)
O(7)-Gd(2)-O(1)	68.84(16)	O(35)-Gd(5)-O(40)	80.76(16)
O(2)-Gd(2)-O(1)	58.91(16)	O(49)-Gd(5)-O(40)	115.30(16)
O(11)-Gd(2)-O(1)	95.72(16)	O(38)-Gd(5)-O(40)	68.53(15)
O(5)-Gd(2)-O(10)	141.14(16)	O(43)-Gd(5)-O(40)	146.78(16)
O(16)-Gd(2)-O(10)	72.63(18)	O(41)-Gd(5)-O(40)	58.40(15)
O(13)-Gd(2)-O(10)	64.50(17)	O(46)-Gd(5)-O(40)	89.51(16)
O(7)-Gd(2)-O(10)	122.24(16)	O(47)-Gd(5)-O(40)	135.88(15)
O(2)-Gd(2)-O(10)	87.42(16)	O(44)-Gd(6)-O(32)#3	79.95(19)
O(11)-Gd(2)-O(10)	57.35(16)	O(44)-Gd(6)-O(47)	85.68(17)
O(1)-Gd(2)-O(10)	140.24(16)	O(32)#3-Gd(6)-O(47)	157.91(18)
O(10)-Gd(3)-O(22)	155.13(17)	O(44)-Gd(6)-O(29)#3	80.08(19)
O(10)-Gd(3)-O(25)	83.19(17)	O(32)#3-Gd(6)-O(29)#3	85.83(18)
O(22)-Gd(3)-O(25)	81.82(17)	O(47)-Gd(6)-O(29)#3	75.18(17)
O(10)-Gd(3)-O(19)	77.02(17)	O(44)-Gd(6)-O(52)	158.98(17)
O(22)-Gd(3)-O(19)	124.82(17)	O(32)#3-Gd(6)-O(52)	118.80(18)
O(25)-Gd(3)-O(19)	98.52(18)	O(47)-Gd(6)-O(52)	78.81(17)
O(10)-Gd(3)-O(14)	121.51(17)	O(29)#3-Gd(6)-O(52)	109.07(18)
O(22)-Gd(3)-O(14)	75.42(16)	O(44)-Gd(6)-O(49)	77.41(17)

O(25)-Gd(3)-O(14)	155.26(17)	O(32)#3-Gd(6)-O(49)	123.10(19)
O(19)-Gd(3)-O(14)	86.83(18)	O(47)-Gd(6)-O(49)	68.96(16)
O(10)-Gd(3)-O(17)	78.52(18)	O(29)#3-Gd(6)-O(49)	138.65(17)
O(22)-Gd(3)-O(17)	79.59(17)	O(52)-Gd(6)-O(49)	83.78(16)
O(25)-Gd(3)-O(17)	80.87(18)	O(44)-Gd(6)-O(50)	97.80(18)
O(19)-Gd(3)-O(17)	155.42(17)	O(32)#3-Gd(6)-O(50)	74.39(17)
O(14)-Gd(3)-O(17)	104.06(18)	O(47)-Gd(6)-O(50)	124.54(16)
O(10)-Gd(3)-O(13)	66.64(17)	O(29)#3-Gd(6)-O(50)	160.15(16)
O(22)-Gd(3)-O(13)	117.53(17)	O(52)-Gd(6)-O(50)	79.84(17)
O(25)-Gd(3)-O(13)	143.32(16)	O(49)-Gd(6)-O(50)	58.24(16)
O(19)-Gd(3)-O(13)	94.63(17)	O(44)-Gd(6)-O(53)	142.50(16)
O(14)-Gd(3)-O(13)	59.09(16)	O(32)#3-Gd(6)-O(53)	70.90(18)
O(17)-Gd(3)-O(13)	73.32(17)	O(47)-Gd(6)-O(53)	113.61(17)
O(10)-Gd(3)-O(20)	123.56(17)	O(29)#3-Gd(6)-O(53)	74.88(17)
O(22)-Gd(3)-O(20)	69.81(16)	O(52)-Gd(6)-O(53)	58.11(16)
O(25)-Gd(3)-O(20)	72.14(16)	O(49)-Gd(6)-O(53)	138.63(15)
O(19)-Gd(3)-O(20)	58.46(16)	O(50)-Gd(6)-O(53)	96.54(16)

Symmetry codes: #1 -x+1,y-1/2,-z+1; #2 -x,y-1/2,-z+2; #3 -x,y+1/2,-z+2; #4 -x+1,y+1/2,-z+1

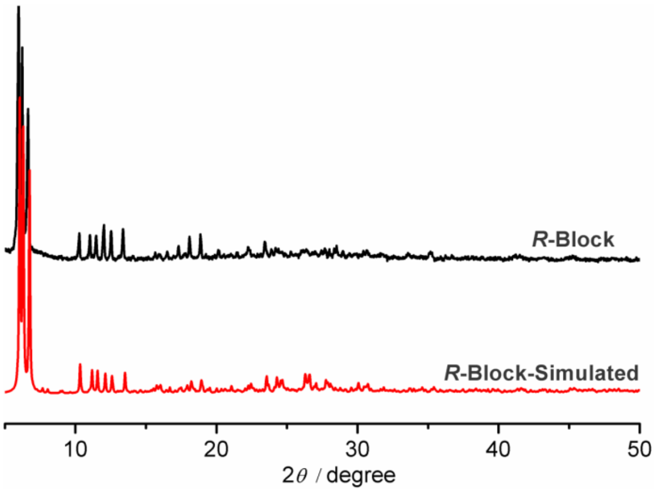

Figure S4. The simulated and experimental PXRD patterns of ***R*-Block**.

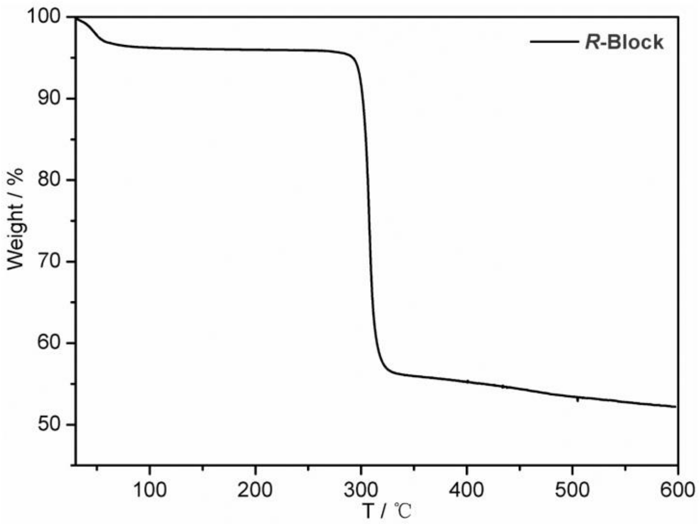

Figure S5. Thermal analysis curves of ***R*-Block**. The weight losses in the temperature 30-100°C are 3.6% for ***R*-Block**, which agree well with the valued expected for the removal of ten lattice water molecules (calcd. 3.4%).

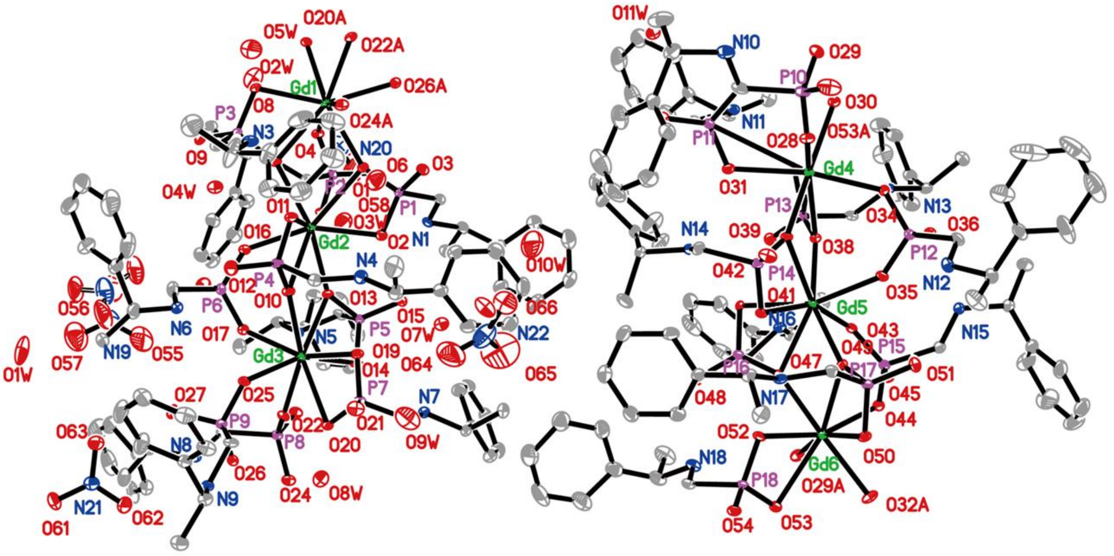

Figure S6. ORTEP view of ***R*-Block** with 30% thermal ellipsoids. All H atoms except those attached to the chiral carbon centers are omitted for clarity. All C atoms except the chiral centers are not labeled.

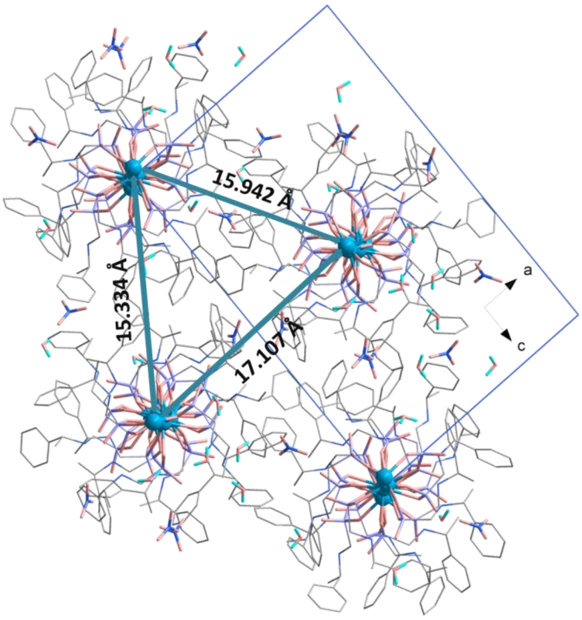

Figure S7. Packing diagrams of structures ***R*-Block** viewed along the crystallographic b-axis. The interchain distances are defined by the distances between the relative 2_1 axis along the b-direction in ***R*-Block**.

Ⅲ. Characterization of superhelices *R*-M and *R*-P

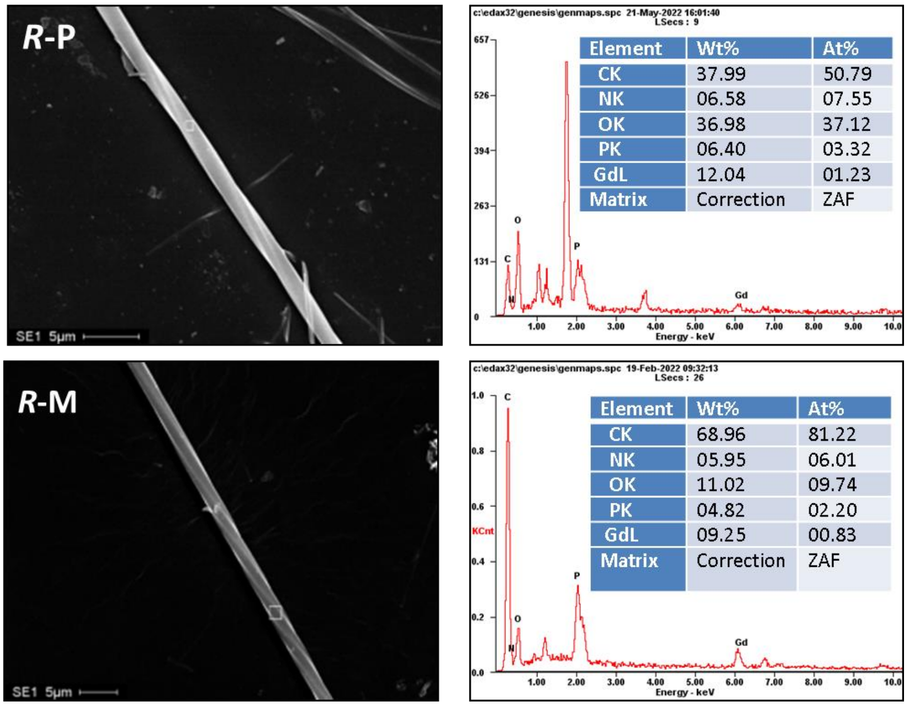

Figure S8. EDS analyses of ***R*-M** and **R-P** at pH 3.2 and pH 3.8, respectively; after hydrothermal reactions of Gd(NO_3)$_3$ and *R*-pempH_2 at 120°C for 24h.

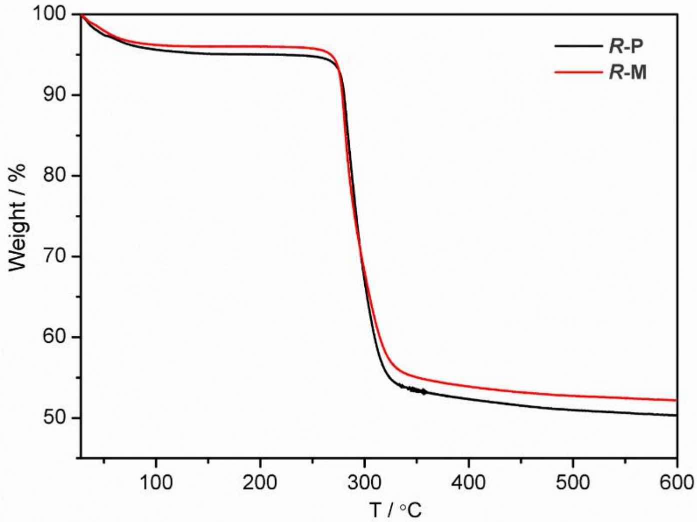

Figure S9. TG analyses of ***R*-M** and **R-P** at pH 3.2 and pH 3.8, respectively; after hydrothermal reactions of Gd(NO_3)$_3$ and *R*-pempH_2 at 120°C for 24h. The weight losses in the temperature 30-120°C are 4.6% for ***R*-P**, and 3.9% for ***R*-M**, which agree well with the valued expected for the removal of two lattice water molecules (calcd. 4.3% for ***R*-P**, 4.2% for ***R*-M**).

Table S3. Elemental analysis of mixture adding different $NaNO_3$ of after hydrothermal reactions of $Gd(NO_3)_3$ and *R*-pempH_2 at 120°C and pH 3.8 for 24h.

	C	H	N	C:N
***R*-Rod**	38.80	5.15	5.03	9.00 0
0.0 eq（***R*-P**）	38.78	5.03	5.05	8.959
0.4eq	38.51	4.76	5.05	8.897
0.8eq	38.30	4.96	5.02	8.901
1.2eq	38.50	5.11	5.13	8.756
1.6eq	37.26	5.09	5.18	8.392
2.0eq (***R*-M'**)	37.94	4.89	5.66	7.820
3.0 eq	37.93	5.02	5.50	8.046
4.0 eq	37.72	4.74	5.53	7.958
***R*-Block**	37.09	4.98	5.90	7.334

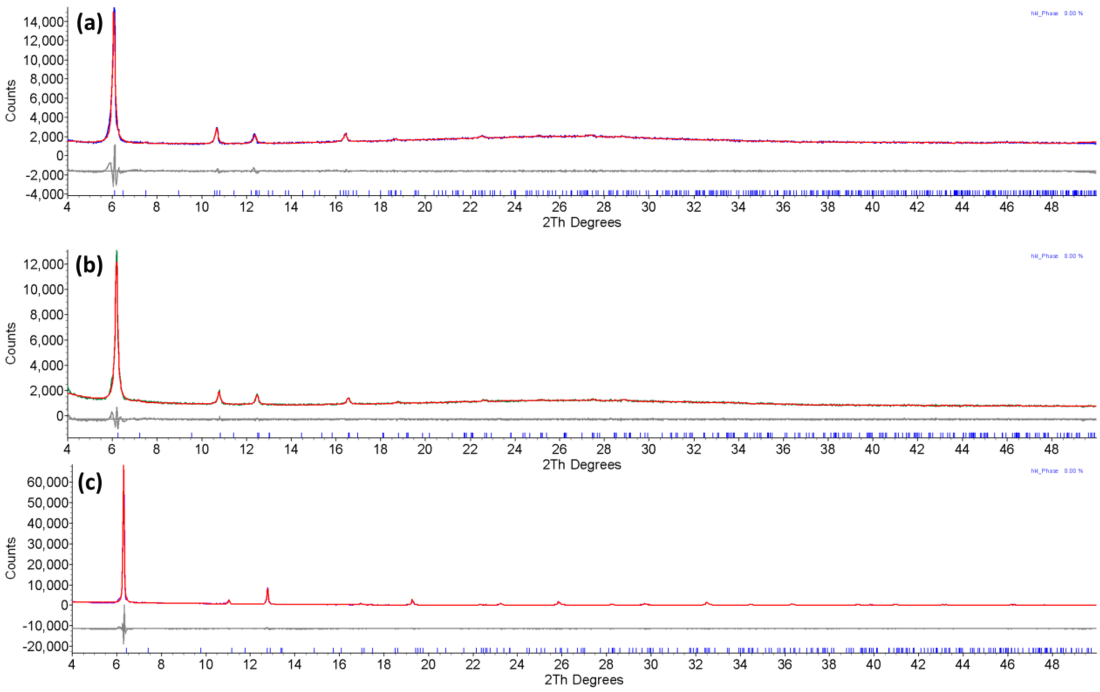

Figure S10. Pawley fit of a powder sample of ***R*-M** (a), ***R*-P** (b), ***R*-Rod** (c) performed using Topas 5.0 program.

Fitted cell parameters for ***R*-M**: $P6_5$, a =16.73 Å, c = 40.30 Å, V = 9767.22Å^3, Rwp = 4.562;

Fitted cell parameters for ***R*-P**: $P6_5$, a =16.34 Å, c = 24.40 Å, V = 5641.98 Å^3, Rwp = 4.122;

Fitted cell parameters for ***R*-Rod**: $P6_5$, a =15.86 Å, c = 24.32 Å, V = 5298.37 Å^3, Rwp = 8.715.

Ⅳ. Salt effect on the chirality inversion of superhelices

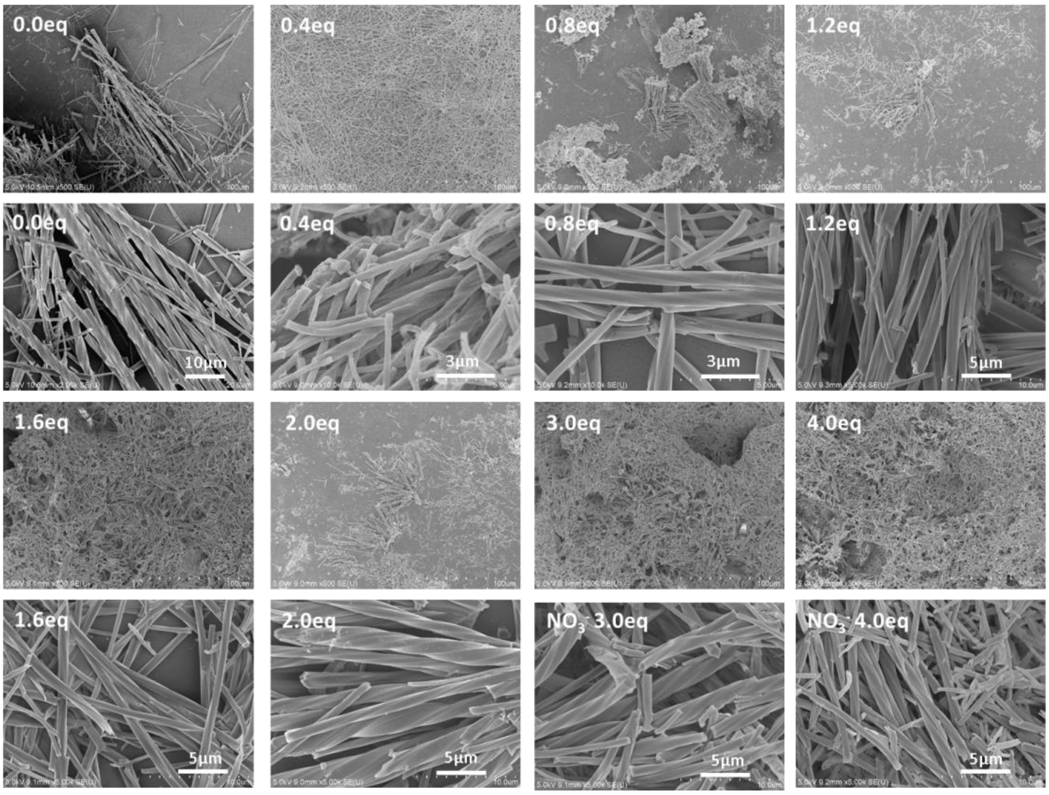

Figure S11. SEM images show the morphology of the product under adding different $NaNO_3$ of mixture after hydrothermal reactions of $Gd(NO_3)_3$ and *R*-pempH$_2$ at 120°C and pH 3.8 for 24 h.

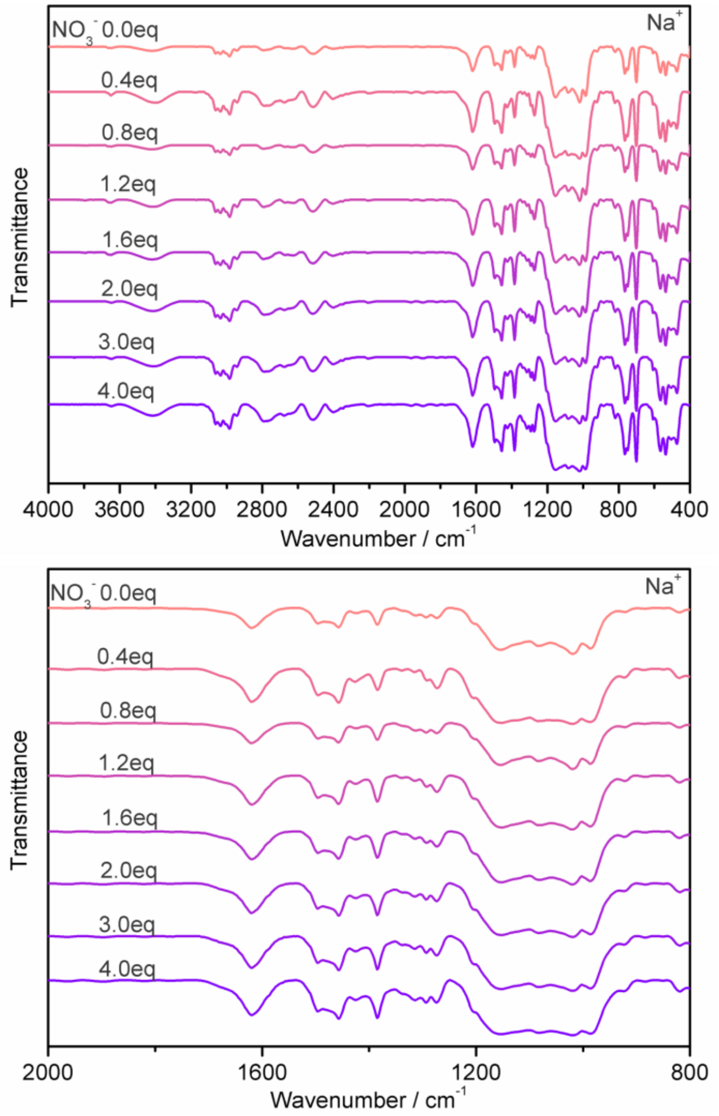

Figure S12. IR spectra of the product under adding different $NaNO_3$ of mixture after hydrothermal reactions of $Gd(NO_3)_3$ and *R*-pempH_2 at 120°C and pH 3.8 for 24h (based on NO_3^-) (Top: 4000-400, Bottom: 2000-800 cm^{-1}).

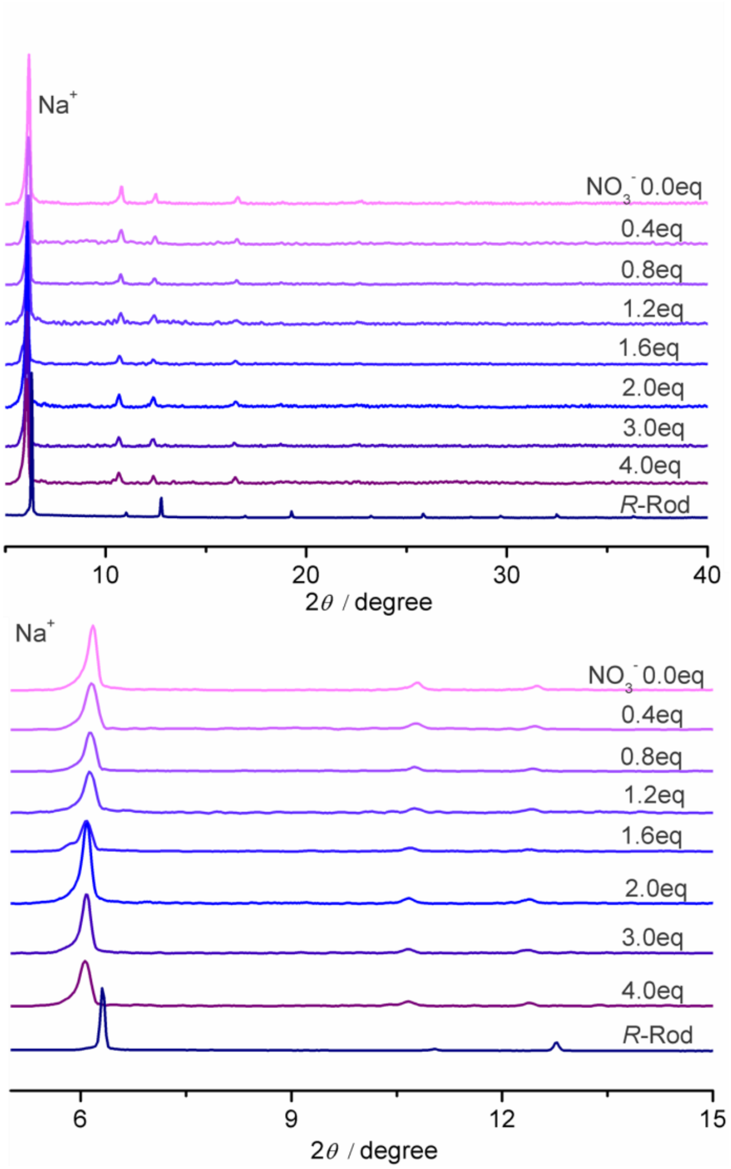

Figure S13. PXRD patterns of the product under adding different $NaNO_3$ of mixture after hydrothermal reactions of $Gd(NO_3)_3$ and *R*-pempH$_2$ at 120°C and pH 3.8 for 24h (based on NO_3^-) (Top: 2θ = 5-40, Bottom: 2θ = 5-15).

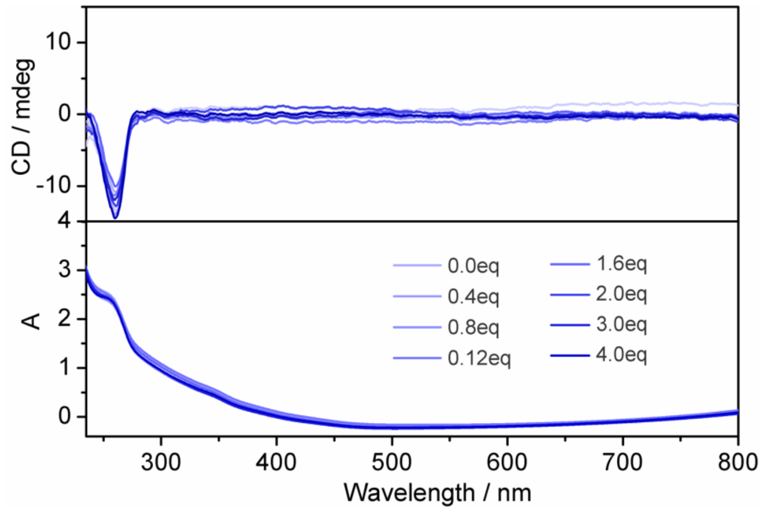

Figure S14. CD spectra of the product under adding different $NaNO_3$ of mixture after hydrothermal reactions of $Gd(NO_3)_3$ and *R*-pempH_2 at 120°C and pH 3.8 for 24h (based on NO_3^-).

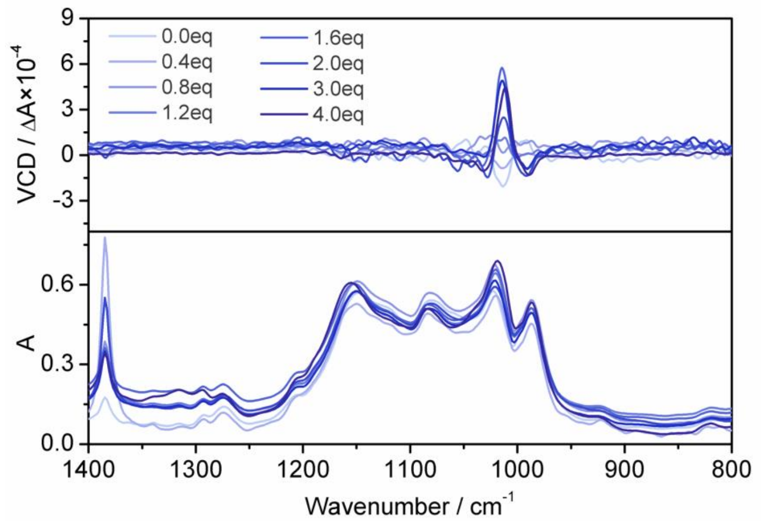

Figure S15. VCD spectra of the product under adding different $NaNO_3$ of mixture after hydrothermal reactions of $Gd(NO_3)_3$ and R-pempH_2 at 120°C and pH 3.8 for 24h (based on NO_3^-).

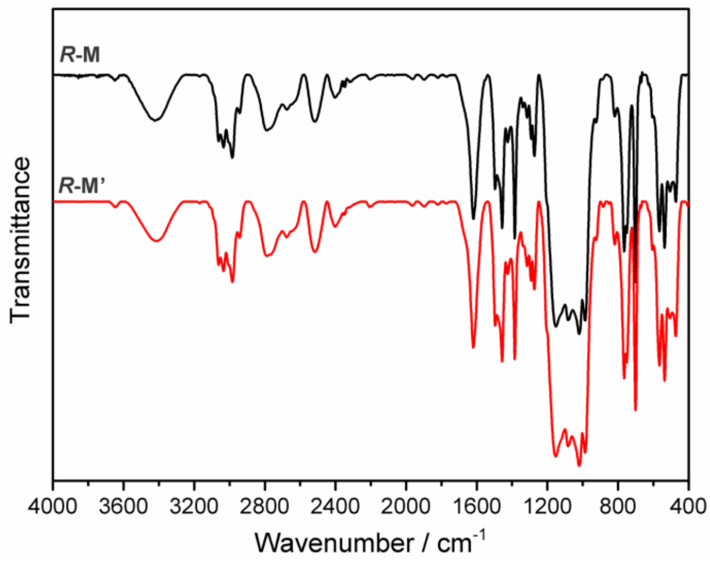

Figure S16. IR spectra of ***R*-M** (pH 3.2) and ***R*-M'** (pH 3.8, NO_3^-: 2.0eq) after hydrothermal reactions of $Gd(NO_3)_3$ and *R*-pempH_2 at 120°C for 24h.

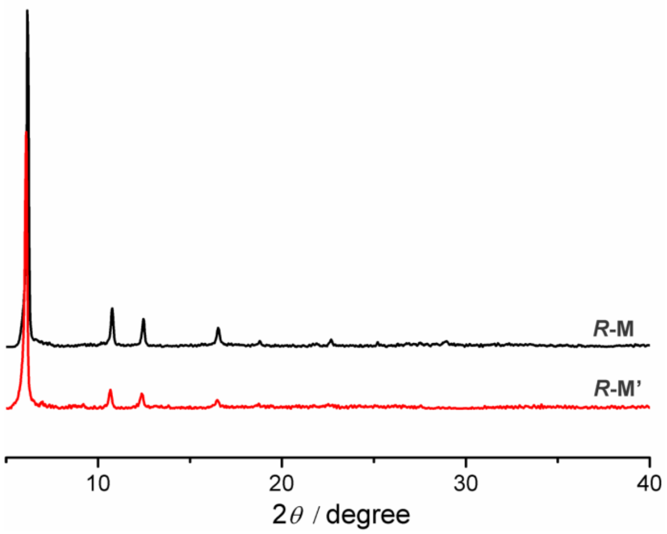

Figure S17. PXRD patterns of ***R*-M** (pH 3.2) and ***R*-M'** (pH 3.8, NO_3^-: 2.0eq) after hydrothermal reactions of $Gd(NO_3)_3$ and *R*-pempH_2 at 120°C for 24h.

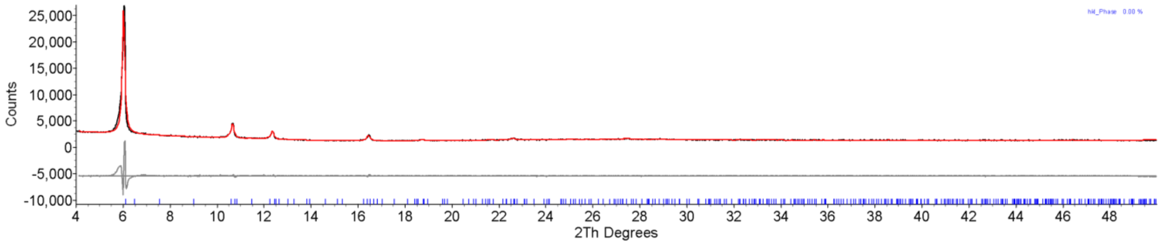

Figure S18. Pawley fit of a powder sample of ***R*-M'** performed using Topas 5.0 program.
Fitted cell parameters: *P*6_5, a =16.82 Å, c =40.37 Å, V =9890.22 Å^3, Rwp = 7.869.

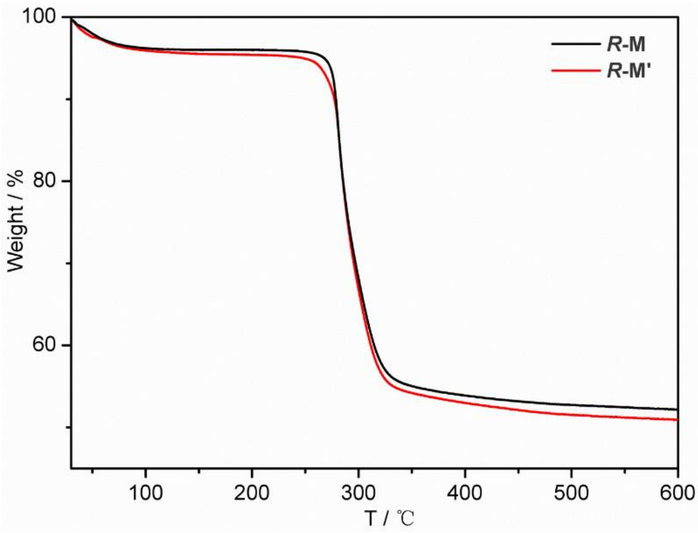

Figure S19. TG analyses of ***R*-M** (pH 3.2) and ***R*-M'** (pH 3.8, NO_3^-: 2.0eq) after hydrothermal reactions of $Gd(NO_3)_3$ and *R*-pempH_2 at 120°C for 24h. The weight losses in the temperature 30-120°C are 3.9% for ***R*-M**, and 4.3% for ***R*-M'**, which agree well with the valued expected for the removal of two lattice water molecules (calcd. 4.2% for ***R*-M**, 4.2% for ***R*-M'**).

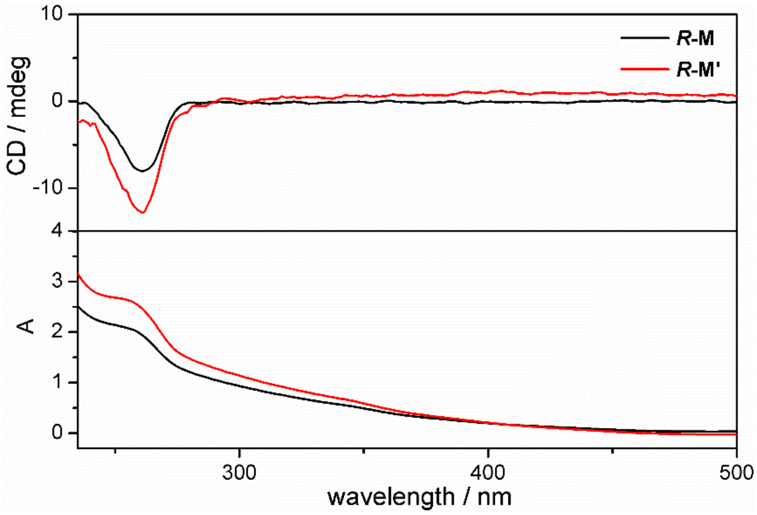

Figure S20. CD spectra of ***R*-M** (pH 3.2) and ***R*-M'** (pH 3.8, NO_3^-: 2.0eq) after hydrothermal reactions of $Gd(NO_3)_3$ and *R*-pempH_2 at 120°C for 24h.

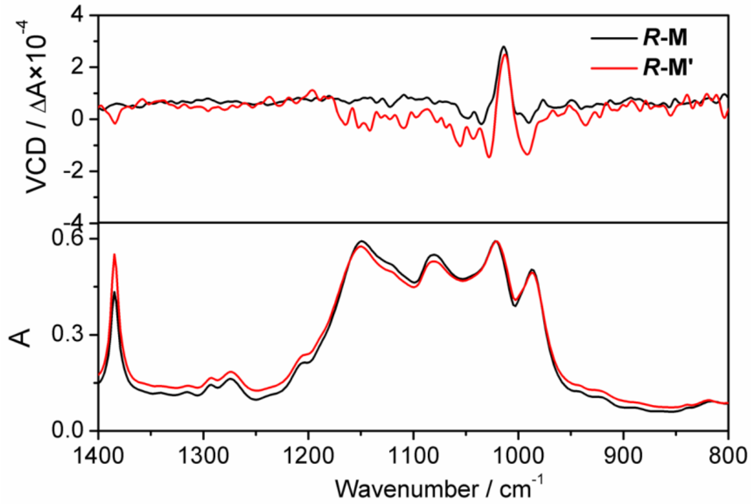

Figure S21. VCD spectra of ***R*-M** (pH 3.2) and ***R*-M'** (pH 3.8, NO_3^-: 2.0eq) after hydrothermal reactions of $Gd(NO_3)_3$ and *R*-pempH_2 at 120°C for 24h.

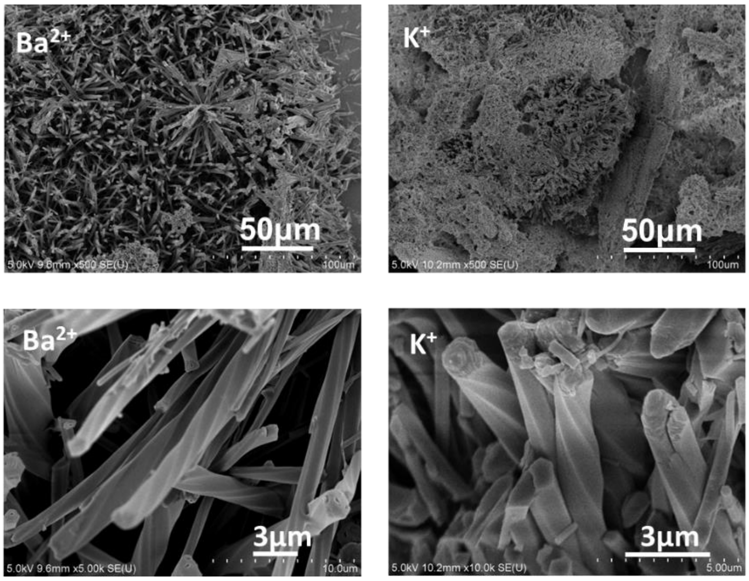

Figure S22. SEM images of products at pH 3.8 adding different nitrates ($M(NO_3)_x$) at 120°C for 24h (2.0eq NO_3^-): $Ba(NO_3)_2$ and KNO_3.

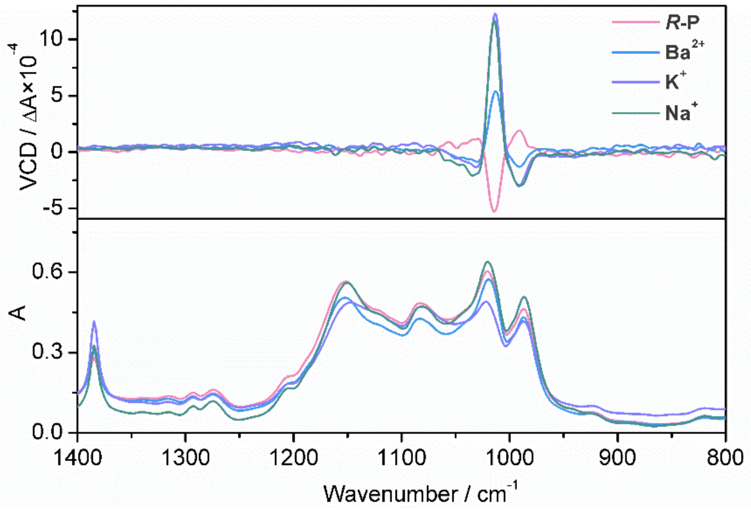

Figure S23. VCD spectra of products at pH 3.8 adding different nitrates ($M(NO_3)_x$) at 120°C for 24h (2.0eq NO_3^-).

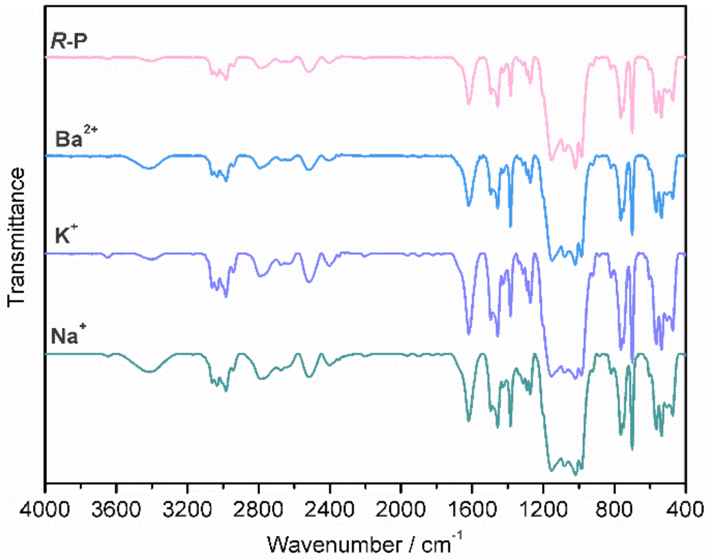

Figure S24. IR spectra of products adding different nitrates ($M(NO_3)_x$) at 120°C and pH 3.8 for 24h (2.0eq NO_3^-).

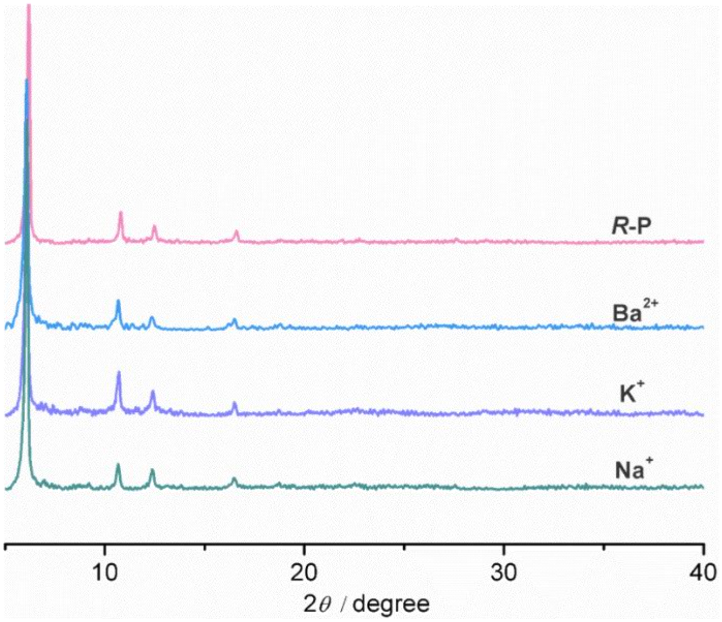

Figure S25. PXRD patterns of products at pH 3.8 adding different nitrates ($M(NO_3)_x$) at 120°C for 24h (2.0eq NO_3^-).

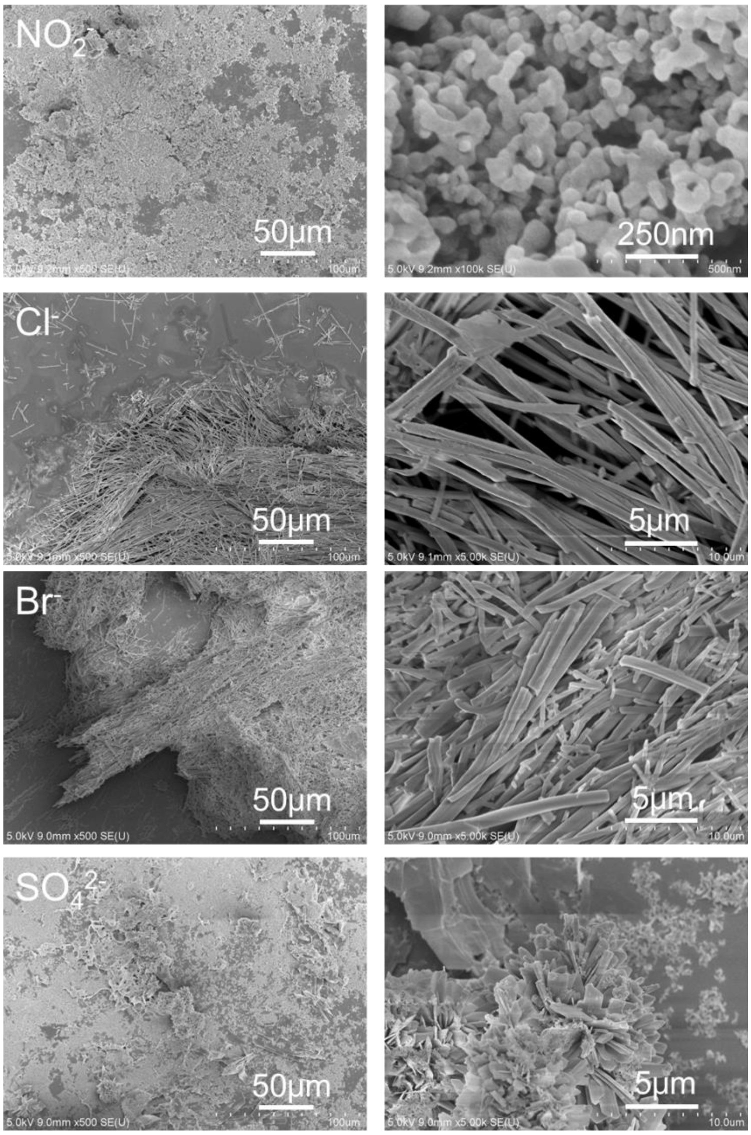

Figure S26. SEM images of products after adding 2.0eq sodium salts NaX (X = Cl, Br, NO_2, SO_4) at 120°C and pH 3.8 for 24h.

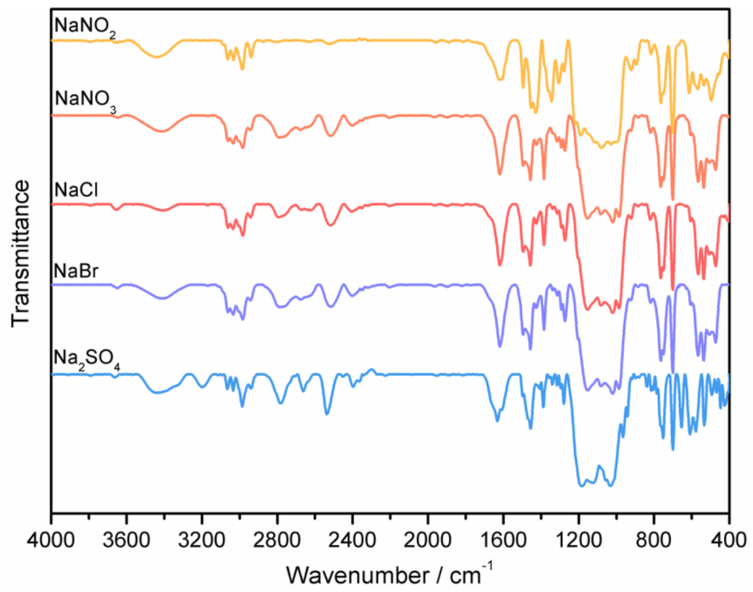

Figure S27. IR spectra of products after adding 2.0eq sodium salts (NaX(X=Cl, Br, NO_2, SO_4)) at 120°C and pH 3.8 for 24h.

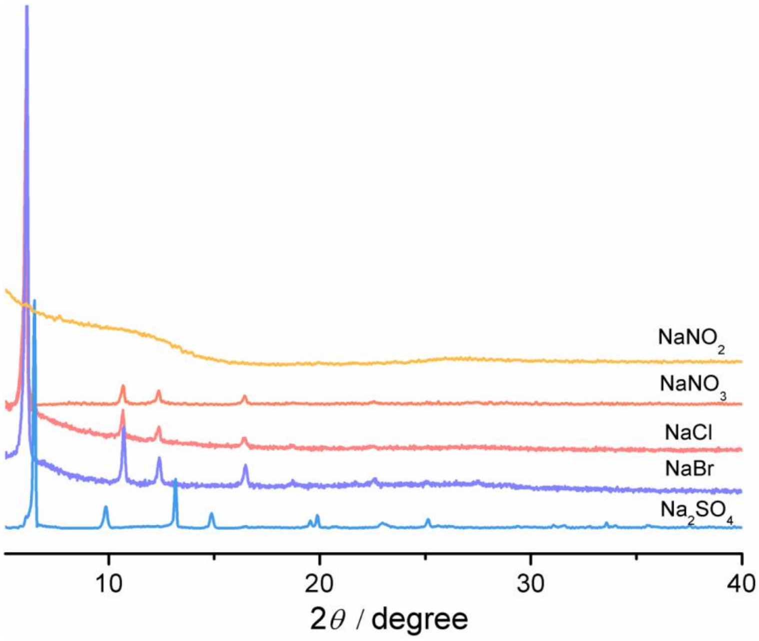

Figure S28. PXRD patterns of products after adding 2.0eq sodium salts (NaX(X=Cl, Br, NO_2, SO_4)) at 120°C and pH 3.8 for 24h.

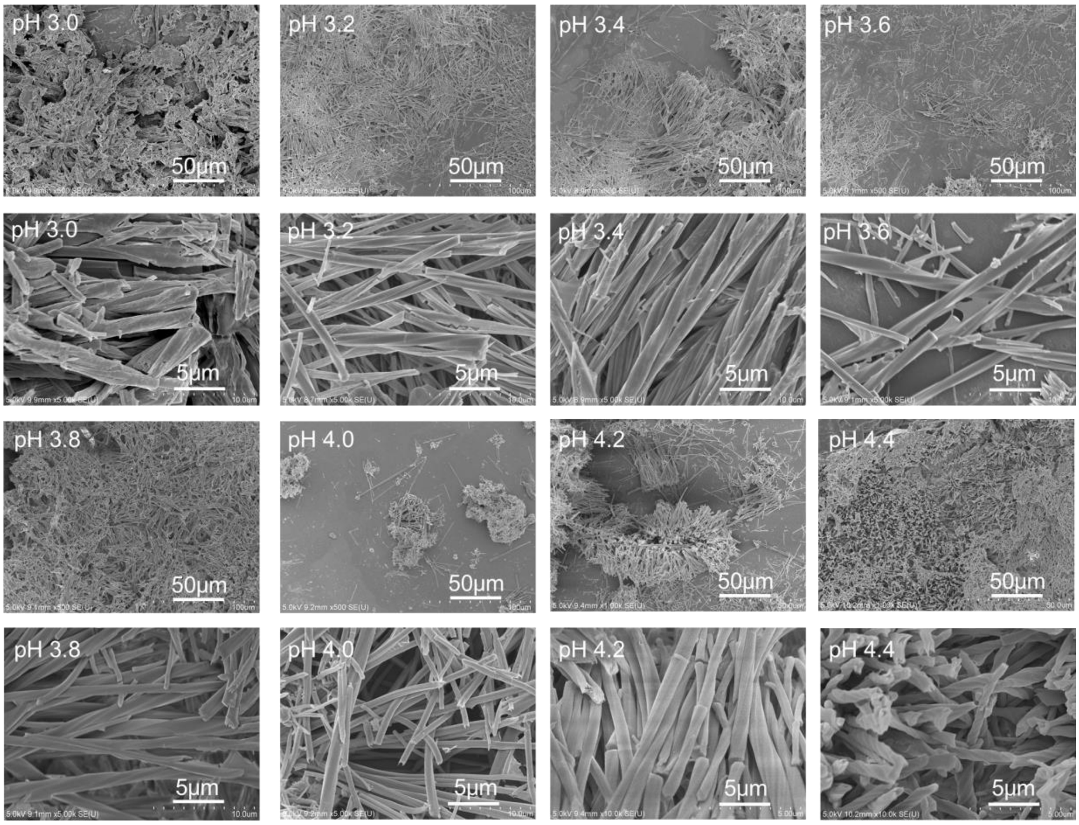

Figure S29. SEM images of products under different pH after adding 2.0eq $NaNO_3$ at 120°C for 24h.

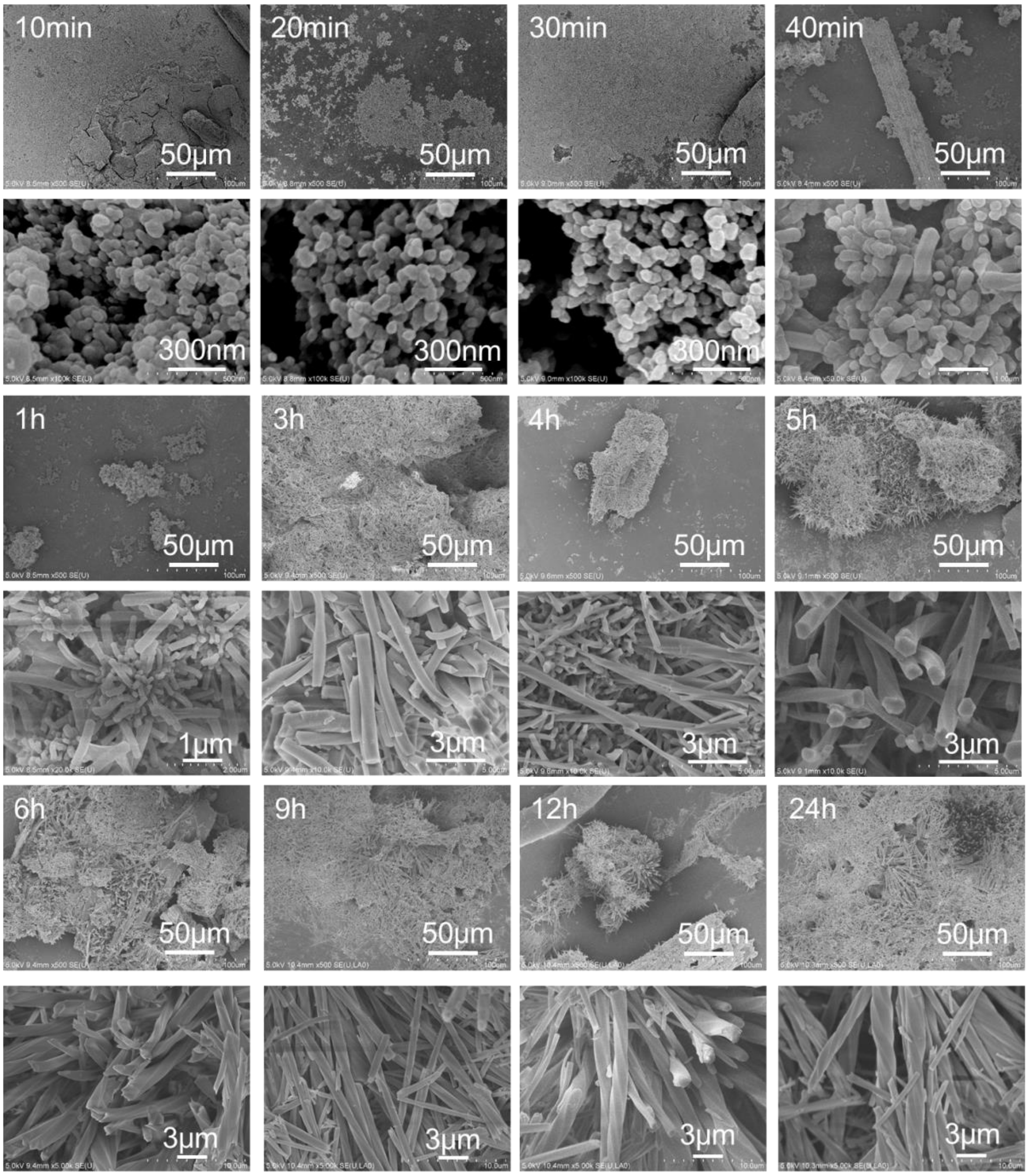

Figure S30. SEM images of products for different period of time at pH 3.8 and 120°C after adding 2.0eq $NaNO_3$.

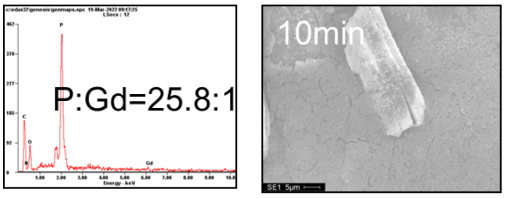

Element	Wt%	At%
CK	55.39	68.45
NK	07.76	08.22
OK	15.62	14.49
PK	17.75	08.50
GdL	03.48	00.33
Matrix	Correction	ZAF

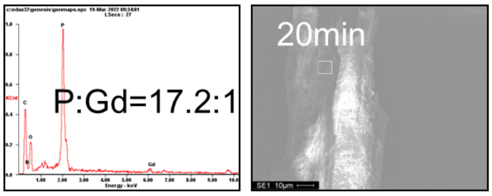

Element	Wt%	At%
CK	55.71	68.41
NK	07.79	08.20
OK	17.29	15.94
PK	14.79	07.04
GdL	04.42	00.41
Matrix	Correction	ZAF

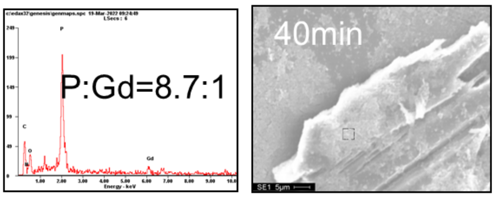

Element	Wt%	At%
CK	47.60	65.09
NK	08.20	09.61
OK	13.46	13.82
PK	19.43	10.30
GdL	11.32	01.18
Matrix	Correction	ZAF

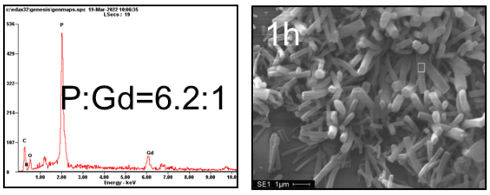

Element	Wt%	At%
CK	43.17	68.42
NK	03.95	05.36
OK	06.85	08.15
PK	25.32	15.56
GdL	20.71	02.51
Matrix	Correction	ZAF

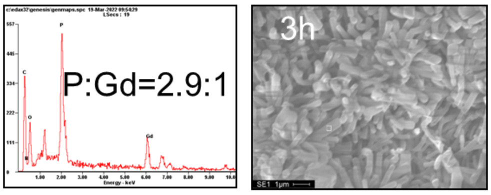

Element	Wt%	At%
CK	48.29	69.67
NK	04.29	05.31
OK	14.89	16.13
PK	11.82	06.61
GdL	20.72	02.28
Matrix	Correction	ZAF

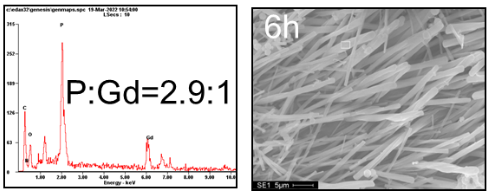

Element	Wt%	At%
CK	42.72	67.39
NK	05.15	06.97
OK	11.34	13.43
PK	14.86	09.09
GdL	25.93	03.12
Matrix	Correction	ZAF

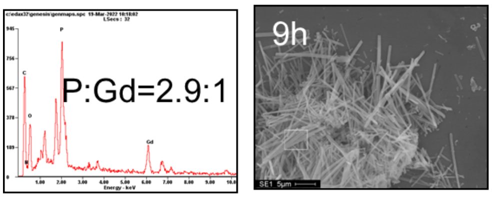

Element	Wt%	At%
CK	49.37	68.96
NK	04.24	05.07
OK	17.24	18.08
PK	10.99	05.95
GdL	18.17	01.94
Matrix	Correction	ZAF

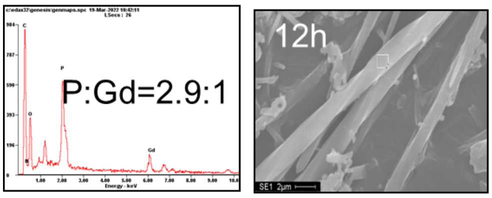

Element	Wt%	At%
CK	57.63	72.85
NK	04.84	05.25
OK	18.16	17.24
PK	07.10	03.48
GdL	12.27	01.18
Matrix	Correction	ZAF

Figure S31. EDS results of products for different period of time at pH 3.8 and 120°C after adding 2.0eq $NaNO_3$.

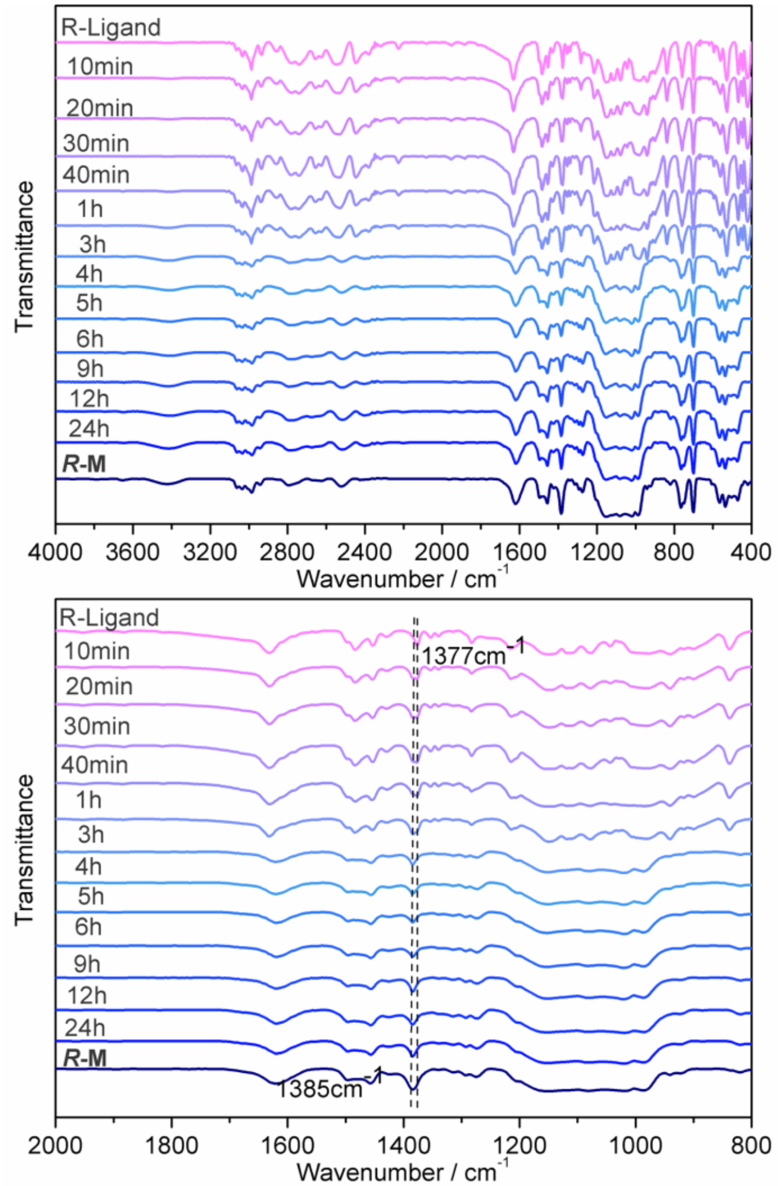

Figure S32. IR spectra of products for different period of time at pH 3.8 and 120°C after adding 2.0eq $NaNO_3$ (Top: 4000-400, Bottom: 2000-800 cm^{-1}).

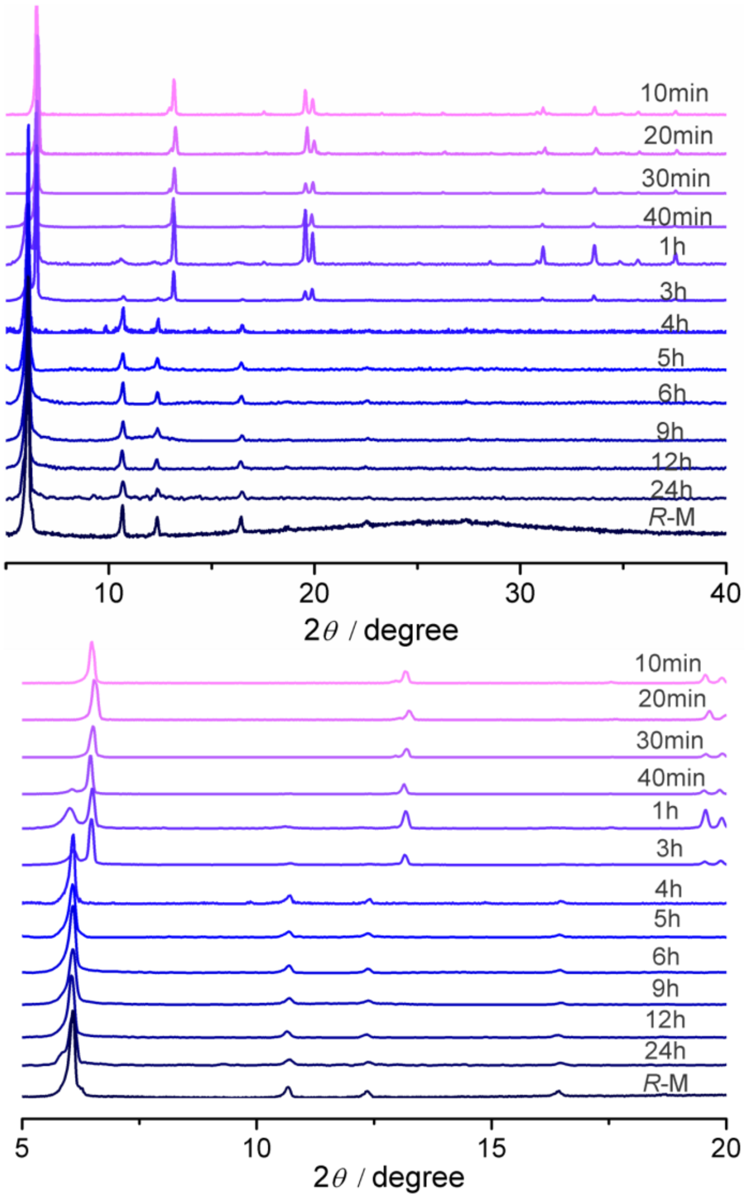

Figure S33. PXRD patterns of products for different periods of time at pH 3.8 and 120°C after adding 2.0eq $NaNO_3$ (Top: 2θ = 5-40 °, Bottom: 2θ = 5-20 °).
